# Interfacial Water Dielectric Constant in the Parallel Direction to Its Surface: A Measuring Technique

**DOI:** 10.1021/acsomega.5c05531

**Published:** 2025-09-02

**Authors:** Omar Teschke, Wyllerson Evaristo Gomes, David Mendez Soares

**Affiliations:** † Laboratório de Nanoestruturas e Interfaces, Instituto de Física Gleb Wataghin, UNICAMP, Campinas 13083-859, Brazil; ‡ Faculdade de Quimica, 217493 Pontificia Universidade Catolica de Campinas, Campinas 13012-970, Brazil

## Abstract

The dielectric constant of interfacial water was recently measured in the normal direction to the water surface; this study showed a significant reduction to a value as low as ε ∼3. Here, a technique that measures parallel to the water surface dielectric constant is described. An asymmetric arrangement of electrodes results in a configuration in which surface and bulk charging effects are separated. The water surface is responsible for the initial fast charge rearrangement. Parallel to the water surface, values as low as ε ≈ 35–40 were measured in agreement with models that show that this ∼50% reduction is associated with the interfacial water confined structure.

## Graphical abstract

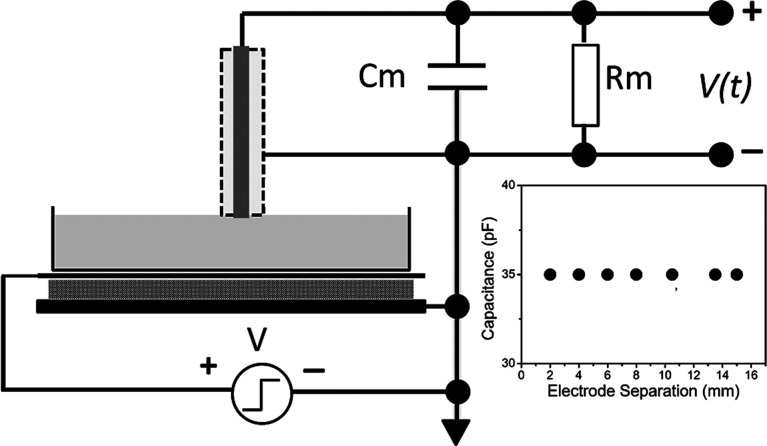

## 1 Introduction

Electrostatic interactions between charged objects in aqueous solution are profoundly influenced by the surrounding water.^1^ In a macroscopic approach, the effect of the water on electrostatic interactions is quantified by means of the static dielectric tensor (ε), which is spatially constant and diagonal in the bulk. Close to an interface, however, the effect of water is more intricate. On an interface, the dielectric constant is anisotropic, which causes the character of interfacial water being distinguishable from bulk water.^2^ Although the dielectric properties of liquid water have been investigated intensively over many decades,^3^ the physical structure of water contacting air is still far from being understood.^4,5^

The capacitance formed by a charged interface and its counterions serves as a sensitive probe of dielectric interfacial effects. It has been known for almost a century that the Gouy–Chapman model overestimates the experimental data of interfacial capacitance, which has been ascribed to a variation of the dielectric constant at the interface.^6^ In the Stern model of the electric double layer, the variation of the dielectric tensor is accounted for by the combination of a length scale and an effective interfacial dielectric constant, reproducing the experimental capacitance.^7^

Theoretical^8^ and experimental studies^9^ have shown that water exhibits layered structuring near surfaces, which suggests that it may form ordered phases under ambient conditions. Such ordered water is generally expected to exhibit small polarizability because of surface-induced alignment of water molecular dipoles, which are then difficult to reorient by applying an electric field.^3,10^ This effect is measured in the vertical direction to the water surface where values as low as ε = 3 were measured.^11^ Given the lack of direct probes for measuring the interfacial water polarizability, most evidence has come from molecular dynamics simulations.^12^ These studies generally predict that the polarizability should be reduced by approximately an order of magnitude,^3,9,10^ but the quantitative accuracy of these predictions is unclear because the same simulation approach struggles to reproduce the known ε for bulk water phases.^13^ Despite extensive studies, the parallel dielectric constant of interfacial water and its depth remain essentially unknown, because measurements are challenging.

This review describes laboratory experiments and the experimental results unveiling interfacial water structures by measuring capacitances of the air/water/electrode periphery contact line intersection associated with radially charging the electrode along the water surface. This water surface radial charging is a proposed method to measure the parallel dielectric constant.

## 2 Experimental Section

The proposed new configuration is schematically shown in Figure 1.

**Figure 1:**
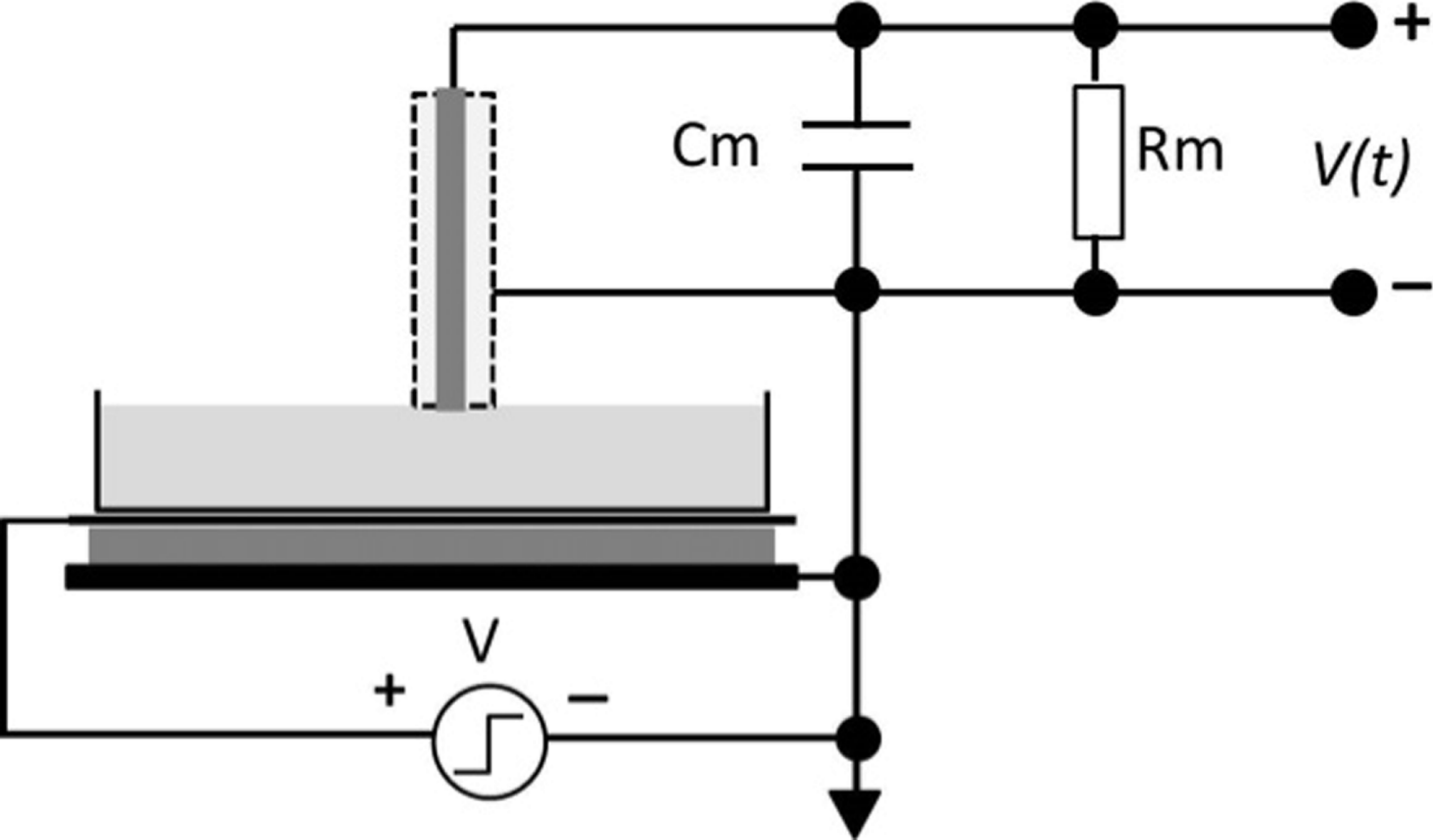
Schematic diagram configuration of the basic circuit for surface charging measurement.

A Petri dish (area *A* = 60 cm^2^) with Milli-Q water (30 or 60 mL) is supported by a circular flat metallic plate, and at the top water surface, a metallic electrode in contact forms the anode. The top metallic electrode is virtually grounded by the integrator formed by Cm and Rm where Cm and Rm are the elements forming the integrator circuit. The water volume in the Petri dish is then in contact with isolated metallic electrodes positioned in the top and bottom water surfaces.

The square wave amplitude potential applied to the bottom electrode (Figure 1) was ±5 V with a periodicity of 0.1 s (10 Hz). The integrating circuit operates as a variable ground electrode switched on and off every 1 s (1 Hz), so this switch grounds the top wire for 0.5 s and then opens the circuit and measures the charge. Initially, there is a fast charging associated with the top capacitor since the top capacitor is *C*_top_ ≪ *C*_bottom_ than the bottom one. The fast short-circuiting of the electrode is performed using a power MFET with τ off for ∼100 ns.

Measurements of the capacitance are taken for voltages applied to the bottom electrode (Figure 1). Detection is made by an integrator circuit and oscilloscope. The method consists of measuring the charge induced by the applied electric potential. The technique essentially displays the charge produced by a fast rise time voltage pulse applied to the water volume. The output voltage *V*(*t*) is given by $V\left(t\right)=\frac{1}{C}\int idt$ , where *i*(*t*) is the induced current varying with time and which is displayed on the oscilloscope. Milli-Q water, DMSO (Merck 99%), Ethanol (Merck 99%), and Methanol (Merck 99%) were used.

## 3 Results

The experimental configuration is shown in Figure 1 where initially the charge rearrangement was tested with an empty cell in order to determine the time response of the measuring circuit. The typical time response is less than 100 ns and corresponds to the time response of the electric circuit associated with air capacitor charging. Then, the container was filled with water and the measured curve is shown in Figure 2. This arrangement is distinct from the classical parallel plate capacitor by the small-sized insulated electrode placed in contact with the upper water surface.

**Figure 2:**
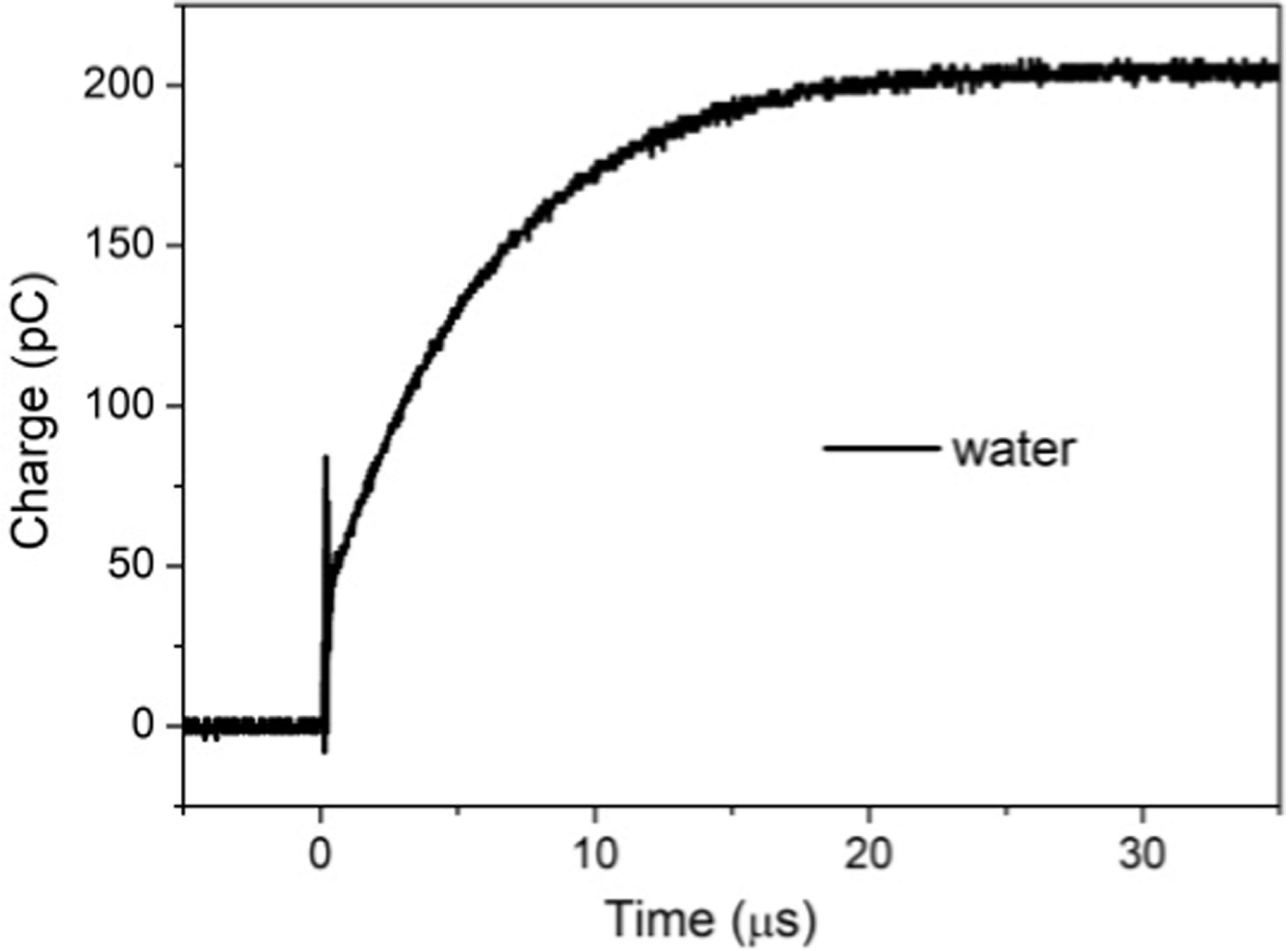
Induced charge measured after a 5 V pulse applied to the bottom electrode (30 mL Milli-Q water in the container).

An initial 40 mV step charging is followed by an exponential increase with time. The final charge is about 200 pC and the charging takes only 20 μs. From the charge versus time curve, the value of the small electrode capacitance is determined. The capacity associated with the charge variation measured value is ∼8 pF for the fast initial charging.

Other interfacial air, solvent, and electrode periphery charging patterns were probed in order to characterize this distinct charging mechanism. Figure 3 shows the charging time dependence profile of methanol (curve a), DMSO (curve b), and ethanol (c).

**Figure 3:**
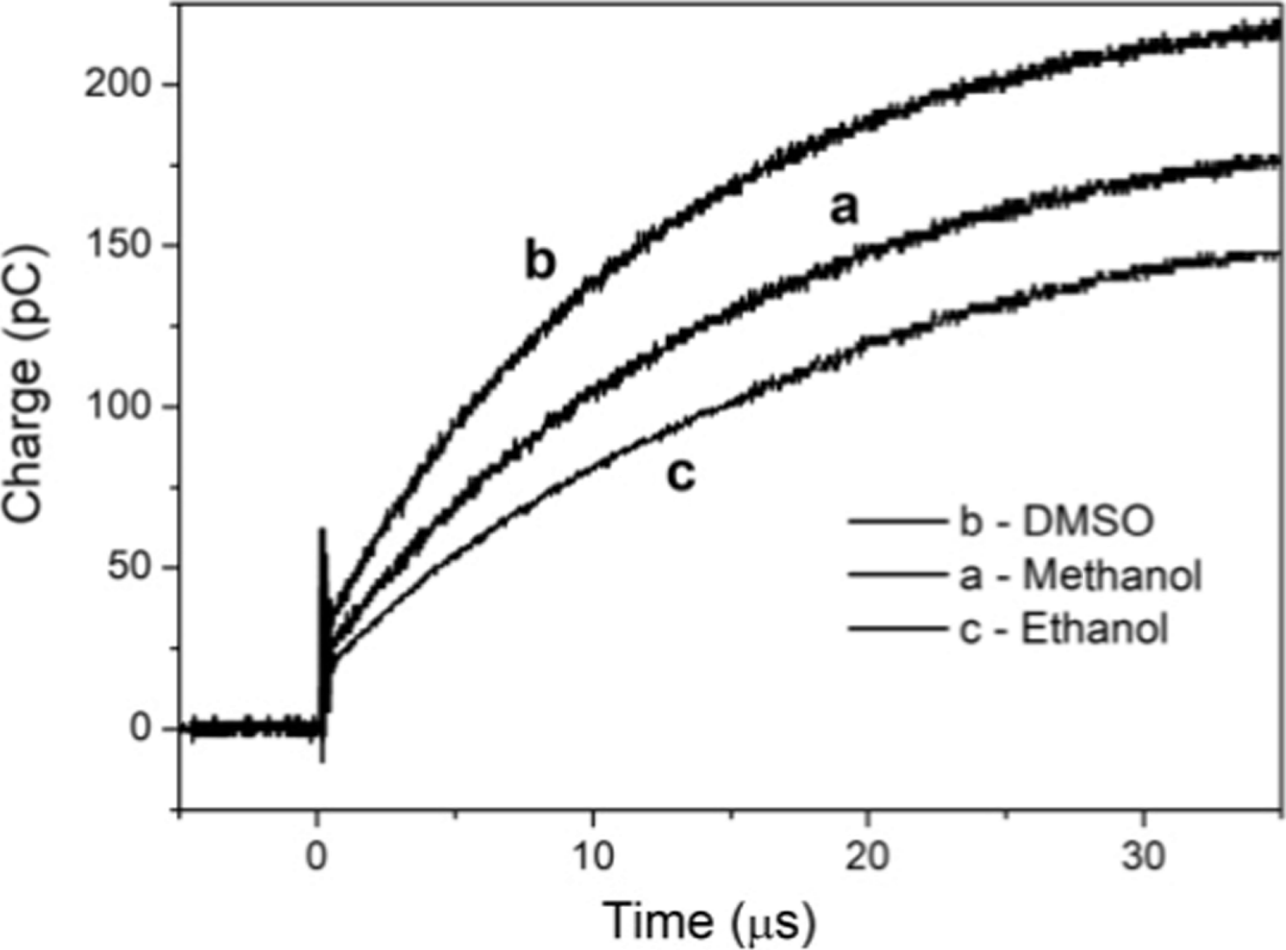
Charging time dependence profile for (a) methanol, (b) DMSO, and (c) ethanol.

In order to characterize the charging mechanism in our configuration (Figure 1), we measured the capacitance for various top electrode radii in contact with a water volume (Figure 4); a linear increase with diameter is observed. This behavior is not observed usually in the capacitance measurements where the capacitance increases with the capacitor area. Then, a distinct behavior was observed in our configuration when compared to parallel plate capacitor configurations.

**Figure 4:**
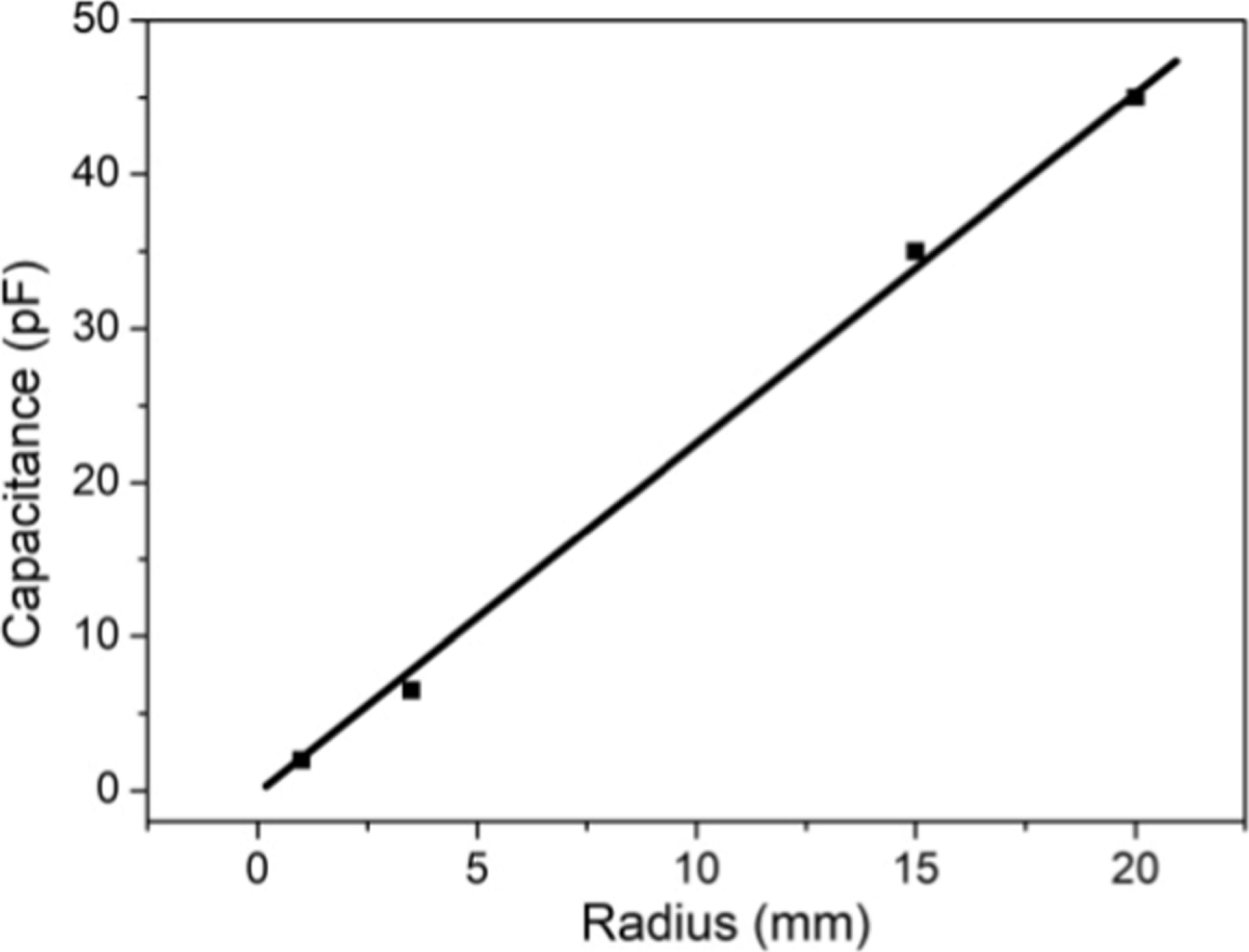
Measured capacitance for various top electrode radius arrangements showing a linear increase with radius variations.

In the next experiment, a new parameter of the electrode arrangement was varied. The separation between the electrodes in contact with a water volume was modified and the results are shown in Figure 5. The measured capacitance for various electrode separations of the four tested small electrode radius arrangements is depicted. Observe that there is no dependence on the value of the electrode separation, i.e., the measured capacitance is independent of electrode separation.

**Figure 5:**
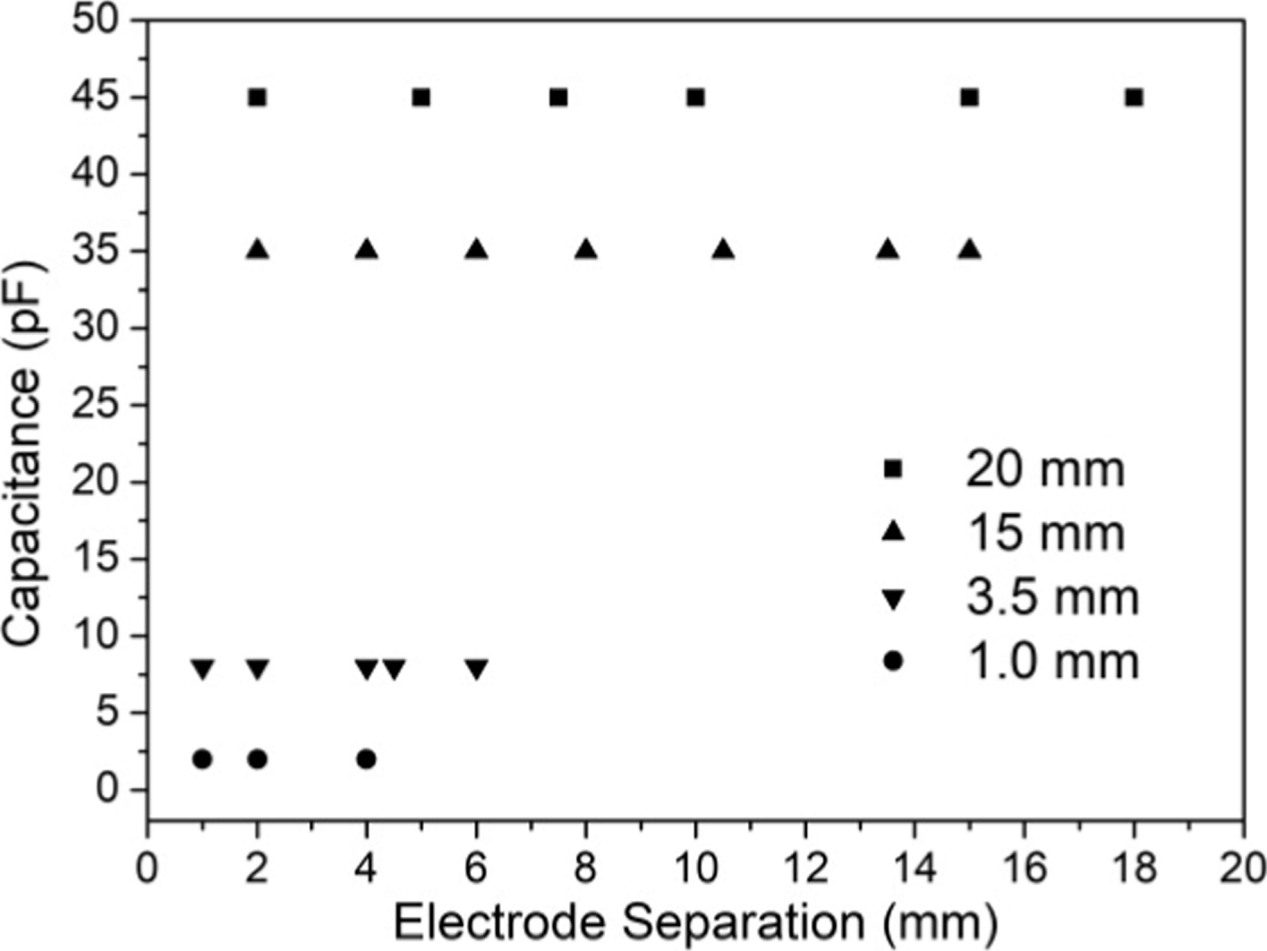
Measured water capacitance vs electrode separations for the tested radii showing that the capacitance is independent of the electrode separation.

These results then suggest that the initial charging of the top small electrode is not associated with the bulk water dielectric as in parallel and equal electrode radius arrangements but to a distinct charging mechanism.

## 4 Discussion

The structure of the water/electrode interface has traditionally been inferred from capacitance measurements by using electrochemical impedance spectroscopy. Many publications have employed this approach^14,15^ with values for the point of zero charge, double-layer thickness, and trends in ion adsorption behavior being reported. In this work, we used a configuration shown in Figure 1 where the two charging mechanisms (surface and volume) can be separated by using a capacitor formed by a large bottom plate and the top small electrode.

### 4.1 Air/Water/Electrode Periphery Contact Line Charging

Common charging capacitor measurements are associated with parallel equal radius electrode configurations which may exhibit a mostly uniform electric field. Here, we have used a new electrode arrangement schematically shown in Figure 6.

**Figure 6:**
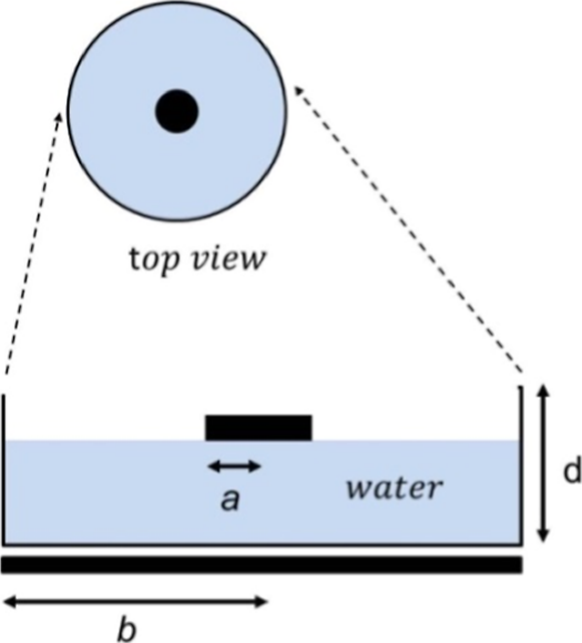
Schematic diagram of the electrode/water/electrode arrangement.

The configuration is formed by a top electrode (with a radius *a*) and a lower electrode (with radius *b*) where *a* ≪ *b*. Let us assume that a positive voltage is applied at the bottom metallic plate, a positive charge will be carried to the top small electrode. The applied step potential at the metallic plate placed below the water surface results in an initial change ΔØ in the potential of the water top surface that charges the top electrode.

The capacitances for disk and ring electrodes with the same diameter were measured in order to determine the (configuration shown in Figure 6) the electrode charging path. The calculated capacitance of a ring electrode with a radius *a* is $C=\frac{Q}{\phi }=4\pi a\varepsilon$ . We have to include the potential associated with the image charge at the metallic electrode surface to calculate the arrangement capacitance. The potential of two charge distributions adds, and the total capacitance is C = 2π*a*ε.

Disk- and ring-shaped electrodes with the same diameter were used, and the measured capacitance shows the same value when measured in contact with the top water surface. For ring- and disk-shaped top electrodes with a diameter of 3.1 cm, the measured capacitance value was 33–35 pF.

Disk and ring electrodes with the same diameter in contact with a water surface show the same capacitance because the contact electrode area with the water surface for both shaped electrodes is the same, i.e., the electrode periphery. Figure 7 shows the results of the charge transport. The charge is deposited at the air/water/electrode periphery contact line, and the negative charge is at the electrode outer surface.

**Figure 7:**
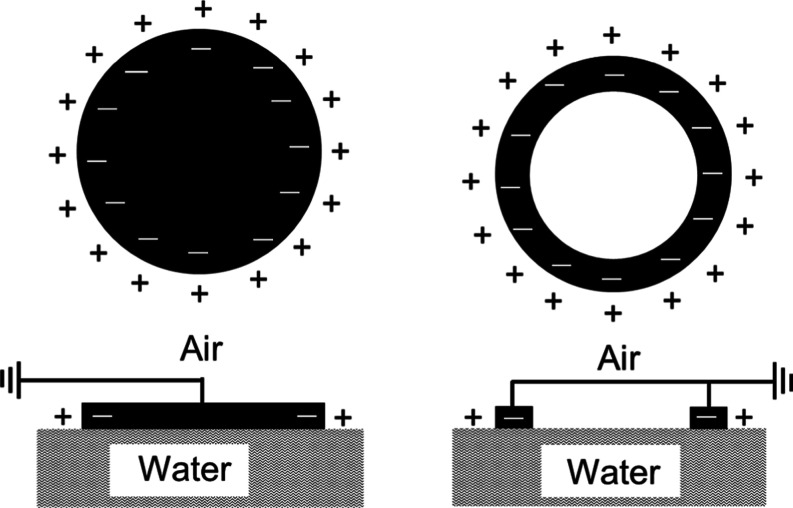
Schematic diagram of the disk and ring electrode charge distribution.

We then assume that there is an applied electric field along the water surface, ending at the edge of the electrode where charges are deposited along the ring and disc perimeter. For an electric field parallel to the water surface, the charge rearrangement will be predominantly carried by the water surface, which is verified by comparing the measured capacitance to the calculated for a ring electrode. Since both disk and ring electrodes have the same capacitance, the contact area of both is its periphery and the charge displacement is along the water surface. Bulk water charge transport results in the disk flat surface charging, and consequently the capacitance of disk and ring electrodes would be distinct.

In conclusion, our results show that the measured capacitance (a) is independent of the electrode separation, (b) dependent linearly on the radius of the electrode, (c) disk electrode and ring electrode with the same radius show the same capacitance, and (d) the measured values of capacitance are given by *C* = 2π*a*ε, where *a* is the electrode radius and ε the dielectric constant. All these facts indicate that the electrode periphery is charged by a surface transport.

### 4.2 Relative Parallel Dielectric Constant

We have calculated the value of the relative parallel dielectric constant (ε_*r*_), using the relation *C* = 2πrε_0_ε_*r*_, for various top electrode radii (*r*) and the results are listed in Table 1.

**Table 1:** Relative Parallel Dielectric Constant Calculated for Different Radius of Top Electrodes

radius (*r*) (mm)	20	15	3.5	3.7	1.5	0.5
dielectric constant (ε_*r*_)	40	41	35	38	36	35
capacitance (pF)	45	35	7	7.8	4	∼1

For a few solvents, the measured relative parallel dielectric constant is shown in Table 2. Bulk dielectric constant and interfacial parallel dielectric constant are presented for water, DMSO, ethanol, and methanol. The interfacial water structure shows a significant reduction (∼50%) in the parallel dielectric constant value compared with that of bulk water. This decrease can be attributed to the ordered structure of interfacial water molecules, which suppress the amplitude of the water dipolar fluctuation. Ethanol and methanol show for the parallel dielectric constant the same value than that of bulk. The interfacial DMSO structure shows like water a significant reduction.

**Table 2:** Measured Longitudinal Dielectric Constant and Its Bulk Values

solution	water	ethanol	methanol	DMSO
bulk	∼80	24.6	32.7	48.0
parallel	35–40	∼20	∼28	∼32

A quantitative description of the structure of a substance such as water is an unsolved problem. Apart from the short-range interactions, one must consider the long-range dipole–dipole forces in order to calculate its dielectric properties. At the interfacial region, the dielectric permittivity will be distinct from the bulk permittivity as measured in this work, since the dipolar interaction will be distinct at the water interface region associated with the formed structure discontinuity at the interface.

### 4.3 Perpendicular Dielectric Constant Spatial Profile Characterizing the Interfacial Region Extension

The measured water surface polarization at the air/water/electrode periphery contact line is associated with the distinct interfacial water structure. To characterize this interfacial region thickness and its structure, we have calculated the perpendicular dielectric constant profiles and we are going to describe a brief review of previous measurements on the normal to the water surface interfacial force vs separation profiles measured using Atomic Force Microscopy.^2^

In order to clarify this technique, an example of a typical force vs separation curve is shown in Figure 8. Using the expression of the dielectric exchange force and the force vs separation measured curve, it is possible to map the interfacial dielectric constant profile. Figure 8 shows from ref 11 the dielectric constant profile and the force vs distance profile that generated this profile. So, the extension of the interfacial layer is associated with the region where ε ≠ 80.

**Figure 8:**
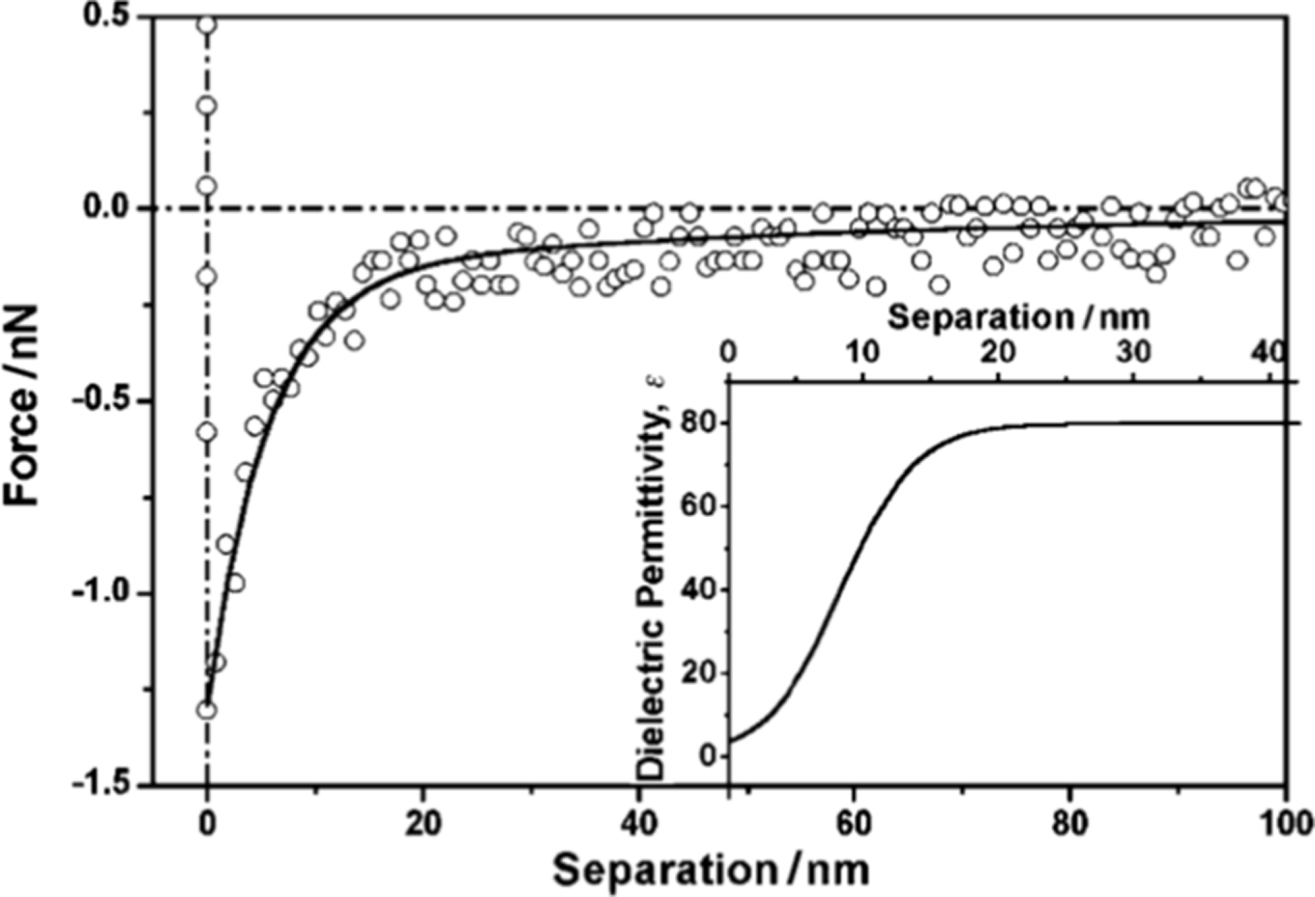
Force vs separation measurements from ref 11. Inset: calculated relative permittivity vs separation curve, which results in the best fitting of the experimental points.

Measurements using interfacial force vs distance profiles show that at water interfaces, there is a suprastructure of water that is not present in the bulk associated with the interfacial electric field, and it is characterized by an interfacial dielectric constant profile (see Figure 8). These structures have a low dielectric constant value as low as ε ∼3.^2,11^

Figure 9 shows steps that were measured at an air/water interface. The range of attractive (vertical arrows) and repulsive (horizontal arrows) forces change as the tip approaches the interface. Closer to the interface, the repulsive force range is ∼1–2 nm and the attractive force has an ∼20 nm extension.

**Figure 9:**
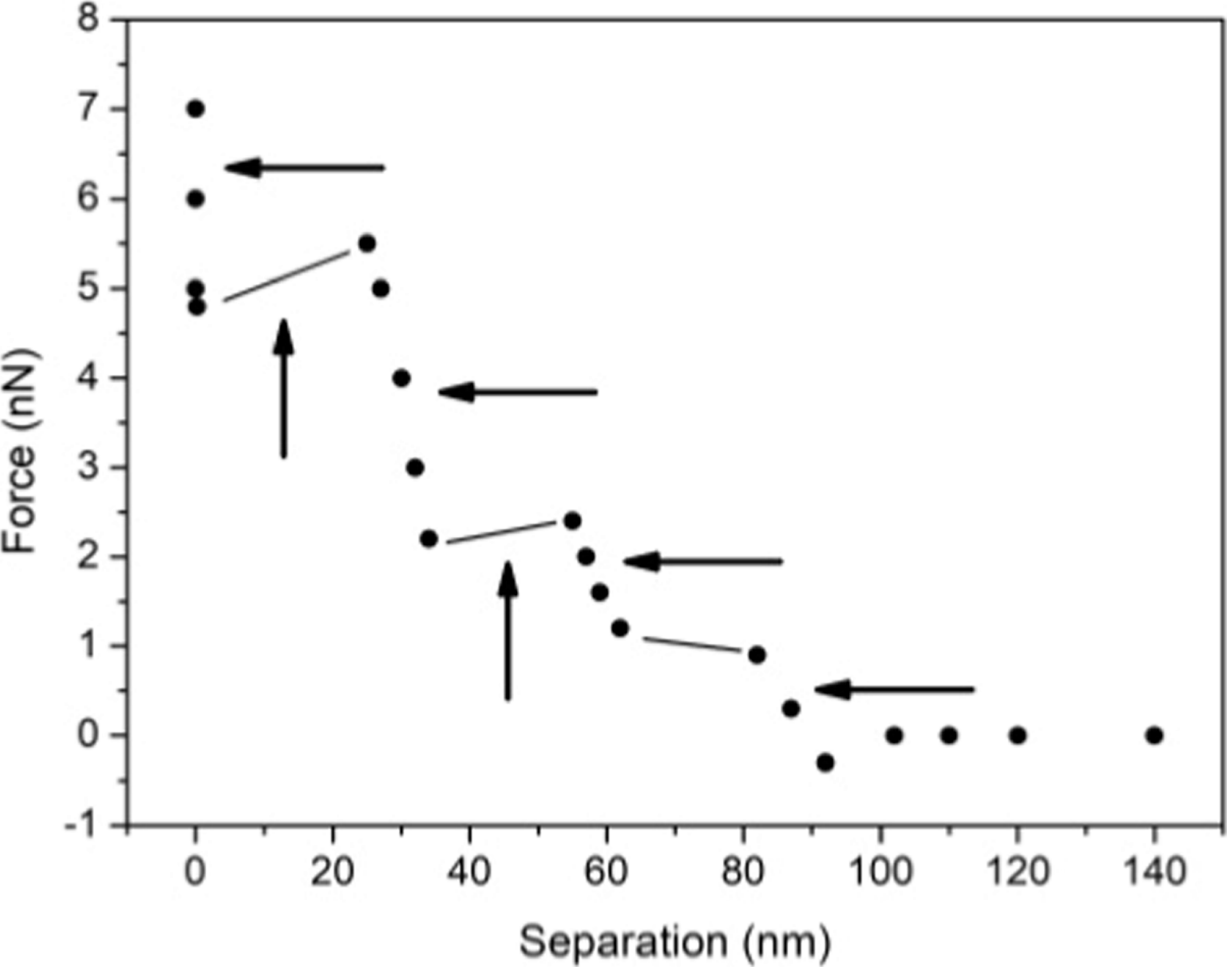
Schematic force vs distance profile for the configuration observed on an air/water interface attached to an air bubble deposited on a PTFE substrate. These profiles characterize the interfacial region clustered structures that are associated with the dielectric constant variable profile interface.

Table 3 depicts a list of cluster profile characteristics measured at the air/water interface. The longest distance to the surface indicates the extension of the interfacial region. The interfacial water structure extends up to ∼93 nm from the water surface in some regions (cluster 4), and in other regions (cluster 2), this structure extends only 8.5 nm from the surface.

**Table 3:** Air/Water Interfacial Profiles Formed at Air Bubbles Deposited on PTFE Substrates^a^

cluster	step thickness (nm)	force amplitude (nN)	surface distance (nm)
1	8.5 8.5 17	1.4 3.3 5.0	34 25.5 17
2	8.5	0.7	8.5
3	25 2	2.3 5	27 2
4	25.5 68	7 14	93.5 68
5	25	4.5	24
6	25	7	24
7	25 42.5	4 5	67.5 42.5
8	34 42.5	3.7 4.5	76.5 42.9

a Clusters extend up to ∼100 nm from the surface (maximum measured thickness of the surface structure layer).

A simple measuring arrangement formed by a small radius top electrode (∼1 mm) and a large radius counter electrode (∼200 mm) isolated by a plastic film, an oscilloscope, and a pulsed power supply is used to measure the parallel water dielectric constant. Here, a new electrical measurement technique is presented, where the water dielectric constant parallel to the surface is measured.

## 5 Conclusions

Water body charge rearrangements in electric fields were measured in order to determine the value of the dielectric constant parallel to the water surface. Application of a step voltage across a water volume in the low-field-strength region (10^–1^ to 10^–3^ V/cm) causes an initial transient, followed by a region of slow charging. This initial charging reflects the polarization charge rearrangement along the water surface. For the air/water interface, the measured longitudinal dielectric constant value ε was ∼35–40, a reduction of ∼50% of the bulk value.
